# Tickborne Encephalitis Virus, Norway and Denmark

**DOI:** 10.3201/eid1207.051567

**Published:** 2006-07

**Authors:** Tone Skarpaas, Irina Golovljova, Sirkka Vene, Unn Ljøstad, Haakon Sjursen, Alexander Plyusnin, Åke Lundkvist

**Affiliations:** * Sørlandet Hospital, Kristiansand, Norway;; † Swedish Institute for Infectious Diseases, Solna, Sweden;; ‡ National Institute for Health Development, Tallinn, Estonia;; § Haukeland University Hospital, Bergen, Norway;; ¶ University of Helsinki, Helsinki, Finland

**Keywords:** tickborne encephalitis, tickborne encephalitis virus, Norway, Denmark, dispatch

## Abstract

Serum from 2 Norwegians with tickborne encephalitis (TBE) (1 of whom was infected in Denmark) and 810 Norwegian ticks were tested for TBE virus (TBEV) RNA by reverse transcription–polymerase chain reaction. Sequencing and phylogenetic analysis were performed. This is the first genome detection of TBEV in serum from Norwegian patients.

Tickborne encephalitis (TBE) is a viral zoonotic disease caused by TBE flavivirus (TBEV). Three subtypes of TBEV have been reported: the European (TBEV-Eu) subtype, transmitted by *Ixodes ricinus* ticks and widely distributed in Europe, and the Siberian (TBEV-Sib) and Far-Eastern (TBEV-FE) subtypes, carried by *I. persulcatus* ticks and present from the Far-East to Baltic countries (*1*). TBE is endemic in Scandinavia along the coastal areas of the Baltic Sea. The first reports of TBE from Sweden, Finland, and Denmark date back to 1954, 1956, and 1963, respectively. The disease was not diagnosed in Norway until 1997 (*2*). Since then, 11 serologically confirmed cases of indigenous human TBE have been reported. A related flavivirus has been isolated in Norway from sheep; it was subsequently analyzed as louping ill virus (LIV), not TBEV (*3*).

In neighboring Denmark, 14 human TBE cases on Bornholm Island were reported and serologically confirmed from 1994 to 2002 (*4*). Recently, both TBEV and LIV have been detected in ticks from Bornholm by reverse transcription–polymerase chain reaction (RT-PCR), although these viruses have not been further characterized genetically (*5*). Antibody tests suggest that human disease in Norway and Denmark is caused by TBEV, but virus has not been isolated from humans in these countries. The aim of this study was to identify and genetically characterize TBEV from Norway.

## The Study

Serum collected before the appearance of TBEV-specific immunoglobulin M (IgM) (acute-phase serum) was available from 2 of 11 TBE patients. The patients, both 38 years of age, included a man from Vest-Agder County, who had not been abroad during the last 4 weeks before disease, and a woman from Hordaland County, who was bitten by a tick on Bornholm Island. Both patients were hospitalized with intensive headache. Results of clinical and neurologic examinations were normal. Their leukocyte counts in cerebrospinal fluid were 87–100/mm^3^. Both patients recovered.

High levels of TBEV IgM and moderate to high levels of TBEV IgG were detected in convalescent-phase sera from both patients by enzyme immunoassay (Enzygnost, Dade Behring, Marburg, Germany).

Ticks were collected by dragging a blanket in the field in areas where patients with TBE had been reported. All collected ticks were unfed. A total of 360 nymphs, adults, and larvae were collected in May and June 2003, and 450 nymphs, adults, and larvae were collected from August 27 to October 8, 2004. Ticks were pooled according to collection site. All pools were stored at –70°C until preparation of tick suspensions.

Acute-phase sera from the 2 patients and 810 ticks (*I. ricinus*) were examined for TBEV. RNA was extracted from serum samples and tick suspensions by using the QIAamp Viral RNA Mini Kit (Qiagen, Germantown, MD, USA) and the TriPure RNA isolation system (Roche Diagnostics, Lewes, UK), respectively. For initial detection of TBEV RNA, the 5´ noncoding region was amplified by nested RT-PCR (*6*), and positive samples were amplified in the coding E protein region (nucleotides [nt] 1323–1765). RT was performed by using MMLV (Moloney murine leukemia virus) RT kit (Invitrogen, Carlsbad, CA, USA) and reverse primer 827R (nt 1777–1800) (Table) according to manufacturer's recommendations. PCR and nested PCR were performed by using primer pairs 283F1 (nt 1233–1255) to 827R1 (nt 1777–1800) and 349F2 (nt 1301–1322) to 814R2 (nt 1766–1787), respectively (Table). Additional details about PCR assays are available from the corresponding author upon request. PCR amplicons were purified and sequenced by using a DNA sequencing kit (ABI Prism, PE Biosystems, Foster City, CA, USA) on a 3100 genetic analyzer (PE Biosystems).

**Table:** Primers used for reverse transcription–polymerase chain reaction

Primer	Sequence (5´→ 3´)
283F1	GAG AC/TC AGA GTG AC/TC GAG GCT GG
827R1	AGG TGG TAC TTG GTT CCA/C TCA AGT
349F2	GTC AAG GCG T/GCT TGT GAG GCA A
814R2	TTC CCT CAA TGT GTG CCA CAG G

The Phylip program package (*7*) was used to analyze the E protein gene sequence data: 500 bootstrap replicates (Phylip's SeqBoot Program) were fed to the Dnadist program (with Kimura's 2-parameter option), distance matrixes were analyzed with the Fitch program (which used the Fitch-Margoliash algorithm), and the bootstrap support values for the tree were calculated with the Consense program . The sequences obtained from GenBank for comparison are listed in the Figure.

**Figure:**
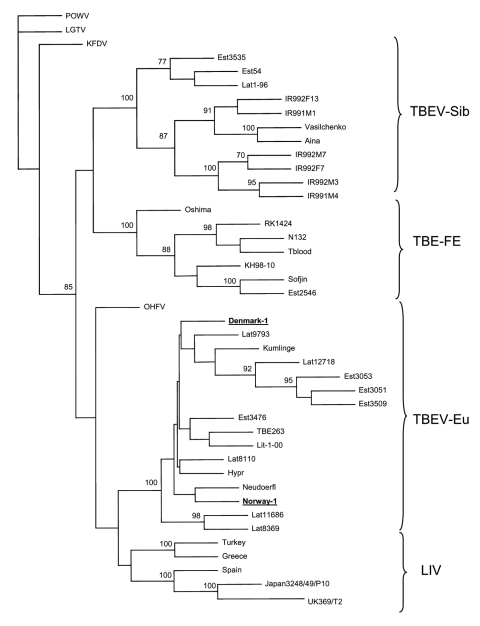
Consensus phylogenetic tree (Fitch-Margoliash) based on the E protein encoding sequence (nucleotides 1323–1765). Only bootstrap support values >70% are shown. Newly identified tickborne encephalitis virus (TBEV) strains from Norway and Denmark are underlined. Abbreviations and accession numbers for viruses and TBEV strains: Powassan virus, POWV, strain LB (L06436); Langat virus, LGTV, strain TP21 (AF253419); Kyasanur forest disease virus, KFDV, (X74111.1); Omsk hemorrhagic fever virus, OHFV, (X66694.1); TBEV, strain IR991M4 (AB049349); TBEV, strain IR992M3 (AB049350); TBEV, strain IR992M7 (AB049351); TBEV, strain IR992F7 (AB049352); TBEV, strain IR992F13 (AB049353); TBEV, strain IR991M1 (AB049348); TBEV, strain Aina (AF091006); TBEV, strain Vasilchenko (AF069066); TBEV, strain Latvia 1–96 (AJ415565); TBEV, strain Oshima (AB022292); TBEV, strain Sofjin (X03870.1); TBEV, strain KH98–10 (AB022297); TBEV, strain Tblood (AF091019); TBEV, strain RK1424 (AF091016); TBEV, strain N132 (AF091013); TBEV, strain 263 (U27491); TBEV, strain Latvia-12718–00 (AJ319586); TBEV, strain Hypr (U39292); TBEV, strain Neudoerfl (U27495); TBEV, strain Latvia-9793–00 (AJ319585); TBEV, strain Latvia-8110–00 (AJ319583); TBEV, strain Kumlinge-A52 (X60286); TBEV, strain Latvia-8369–00 (AJ19584); TBEV, strain Latvia-11686–00 (AJ319582); TBEV, strain Lithuania-1–00 (AJ414703); louping Ill virus (LIV), strain UK369/T2 (Y07863.1); LIV, strain Turkey (LO1265); LIV, strain Greece (X77732); LIV, strain Spain (X77470.1); LIV, strain Japan 3248/49/P10 (M94956.1).

From 360 ticks collected in 2003, only 1 pool (10 ticks) was positive by amplification of the 5´ noncoding region; similarly, only 1 pool of 450 ticks collected in 2004 was RT-PCR-positive. From the positive tick pool in 2004, we sequenced 179 nt of the highly conserved region in 5´ region of TBEV, enough to prove that the virus was TBEV, not LIV. No material from the tick pools was left to attempt virus isolation. Thus, the overall virus prevalence in ticks was 0.3% in 2003 and 0.2% in 2004, if we assume that only 1 tick in the positive pool was infected.

A partial E gene sequence (443 bp) was recovered from the 2 human samples. The corresponding TBEV strains were designated as Norway-1 and Denmark-1. The identity on the nucleotide level between these sequences was 98.6%, and they showed 97.2%–99.0% identity to other TBEV strains within the TBEV-Eu subtype. The levels of sequence identity to strains belonging to TBEV-FE and TBEV-Sib subtypes were 81.4%–83.0% and 83.5%–86.2%, respectively. Phylogenetic analysis of these sequences showed that they belong to the TBEV-Eu subtype (Figure), which does not show clear, separated lineages correlating to geographic regions. The Norwegian TBEV strain clustered together with strain Neudoerfl isolated in Austria, and the Denmark-1 strain clustered together with the group of strains from Latvia, Finland (Kumlinge), and Estonia, albeit bootstrap supports of these clusterings were below the widely accepted confidence limit, 70%. The sequence identity between strains Denmark-1 and Neudoerfl was 99.0%; between strains Norway-1 and Latvia9783, the sequence identity was 98.7%.

## Conclusions

This is the first report of TBEV RNA in serum from Norwegian patients. One of the 2 patients was infected in Vest-Agder County in Norway, and the other on Bornholm Island, Denmark. Genetic analysis showed that the Norwegian and Danish strains belong to the TBEV-Eu subtype. Although the sequences of Norway-1 and Denmark-1 strains showed the highest level of identity to the corresponding sequences of TBEV-Eu subtype, they were distinguishable from each other and also from the sequences of TBEV-Eu strains characterized previously.

In TBE-endemic areas in Europe and on Bornholm Island, 0.5%–5% of ticks are infected with TBEV (*5*). In Norway, where TBE is a rare disease, the prevalence is lower (0.2%–0.3%). In Denmark TBEV has been detected in ticks by RT-PCR (*5*), but to our knowledge, no reports of TBEV findings in Danish patients exist.

The emergence of TBE in Norway in the 1990s poses the question of whether these new endemic foci have become truly established recently or have remained unnoticed because of underdiagnosis. Although the northern spread of TBEV due to climate changes has been predicted (*8*), other factors such as rates of contact between ticks and humans, abundance of ticks, and their amplifying hosts may play a role in TBE epidemiology. Further monitoring of the TBE situation in Norway both in patients and nature is needed to establish guidelines for preventive measures and vaccination programs in TBE-endemic areas.

We report the first genome detection and characterization of TBEV from persons with TBE in Norway and Denmark. Our results showed that the Norwegian and Danish strains clustered with earlier reported strains of the TBEV-Eu subtype.
